# Gastro-Jejunal Ileal Interposition with Bipartition: A Salvage Procedure for Severe Protein-Energy Malnutrition After Transit Bipartition

**DOI:** 10.1007/s11695-025-07825-5

**Published:** 2025-04-09

**Authors:** Tugrul Demirel, Ulku Korkmaz, Surendra Ugale

**Affiliations:** 1 Trakya University, Edirne, Turkey; 2 Kirloskar Hospital, Hyderabad, India

**Keywords:** Bariatric surgery, Malabsorption, Gastrojejunal ileal interposition, Transit bipartition, Protein energy malnutrition, Intractable diarrhea

## Abstract

**Background:**

Intractable diarrhea or excess weight loss associated with protein-energy malnutrition (PEM) can occur after Transit Bipartition (TB). This study evaluates the effect of transposing the alimentary limb to the proximal intestines.

**Methods:**

Between 2017 and 2024, ten patients with malnutrition and diarrhea underwent Gastro-Jejunal Ileal Interposition (GJIB) surgery after TB. We prospectively monitored protein-energy malnutrition postoperatively and retrospectively analyzed demographic data, laboratory findings, and anthropometric measurements. Gastric transit scintigraphy was performed on symptomatic and asymptomatic patients to evaluate gastric evacuation diversity between the pylorus and the gastro-ileostomy.

**Results:**

Ten patients (male/female, 6/4) were operated on. The preoperative mean age was 49.4 ± 9.19 years. The mean body mass index (BMI) was 22.19 ± 1.13 kg/m^2^, the mean excess BMI loss (%EBMIL) percentage was 123.26 ± 14.85%, and the total weight loss percentage (%TWL) was 42.35 ± 0.33. Eighty percent of food passed through the gastroileostomy in all patients. The mean follow-up period was 50.56 ± 57.28 months.

Postoperatively, the mean BMI increased to 28.16 ± 2.2 kg/m^2^ (*p* = 0.001), %EBMIL decreased to 79.88 ± 21.53% (*p* = 0.001), and %TWL decreased to 27.31 ± 10.1. Albumin levels rose from a median of 2.1 mg/dl to an average of 3.8 ± 0.78 mg/dl (*p* = 0.001), and stool frequency decreased from 11.56 ± 0.71 to 2.1 ± 2.12 per day (*p* = 0.001). The excluded bowel length percentage (Exl.B%) decreased significantly from 72.4 ± 3.18% to 12.3 ± 1.99% after conversion (*p* = 0.005). All patients were diabetic before and had remission after TB. Glycemic control was preserved after the conversion, with a median HbA1c of 5.4% compared to 5.8% before conversion.

**Conclusions:**

GJIB may be a viable revision procedure for resolving PEM and related complications without compromising the metabolic benefits of the initial surgery on diabetes resolution by decreasing the Exl.B%.

**Graphical Abstract:**

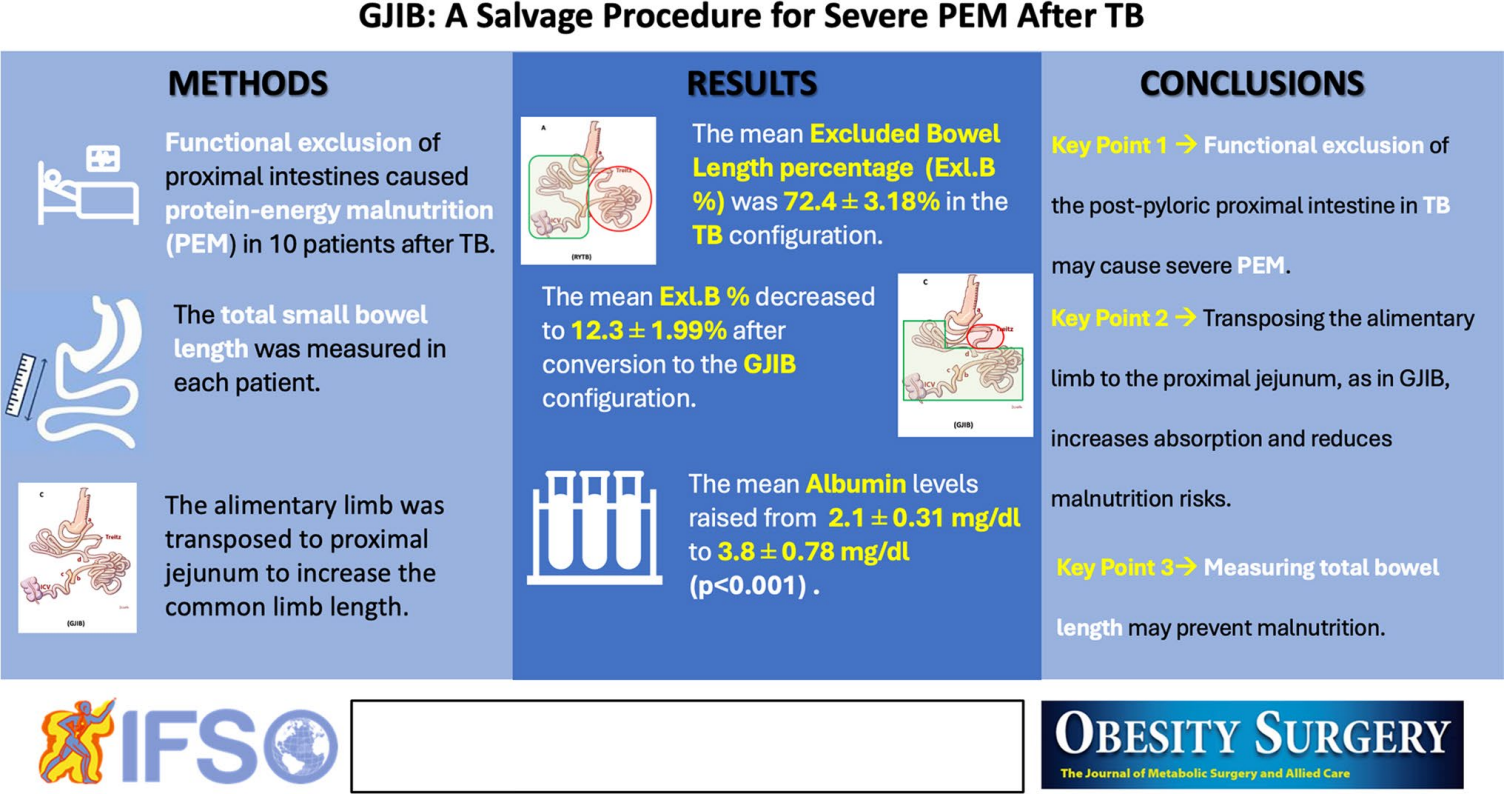

## Introduction

Bariatric surgical procedures range from volume-restrictive techniques such as sleeve gastrectomy to combined techniques with different degrees of malabsorption, as mild as the Roux-n-y Gastric Bypass (RYGB) and as potent as the Biliopancreatic Diversion with Duodenal Switch (BPD/DS) and their variations. Protein-energy malnutrition (PEM) and excess weight loss, related to diarrhea or not, are serious concerns in bariatric surgery. The armamentarium of metabolic surgical procedures continues to expand with “less” or “non” malabsorptive alternatives that use well-known neurohormonal mechanisms such as Duodenoileal Interposition with Diverted Sleeve Gastrectomy (DII-SG) [1] and Transit Bipartition [2] rather than gross intestinal bypasses or strict restrictions of volume or anastomotic diameters.

The Roux-n-y (RYTB) and the one anastomotic configuration (OATB) of Transit Bipartition do not anatomically exclude intestinal segments. Thus, they notably ensure food contact with the foregut, potentially mitigating severe protein-energy malnutrition. However, unexpected severe protein-energy malnutrition with undesired weight loss and stubborn diarrhea was reported in patients with TB [3–6].

The reported treatments for this potentially catastrophic situation were disconnection of the gastroileostomy [3, 4] and elongation of the common limb in the alimentary limb’s expanse [5].

This paper suggests interposing the ileal segment into the proximal jejunum, increasing the absorptive length of the common channel, not at the expense of the alimentary limb, through a new surgical technique, the *Gastrojejunal Ileal Interposition with Bipartition* (GJIB), for treating severe protein-energy malnutrition or intractable diarrhea mimicking malabsorption in TB.

## Methods

This retrospective study’s cohort consisted of ten RYTB and OATB patients who had been converted to GJIB for intractable diarrhea or excess weight loss associated with protein-energy malnutrition (PEM). Six of them were operated on in our center.

All procedures performed in this study involving human participants followed the ethical standards of the institutional and national research committee and the 1964 Helsinki Declaration and its later amendments or comparable ethical standards. The local ethics committee approved this study. All patients gave written informed consent before the conversion surgery.

### Preoperative Evaluation

Patients were admitted with stubborn symptoms of PEM with fatigue, diarrhea, increased appetite, and intractable weight loss. Patients had a typical course in the early postoperative months without signs of PEM like excessive weight loss or intractable diarrhea (Table 1). The patients reported the symptoms of fatigue and increased appetite as self-observations in peer-to-peer follow-ups. Liquid stools with tenesmus were accepted as diarrhea when the frequency was over three times per day, and patients were told to track the daily frequencies while being supported with diet modifications and medical treatments. Patients’ weight status was tracked at follow-ups in outpatient clinic visits. Oral nutritional support was administered for protein, vitamins, and minerals. The surgery decision for conversion was given only when diarrhea and weight loss were intractable with constantly decreasing albumin despite medical and nutritional support. A thorough history of each patient’s stool frequencies, dietary habits, and daily living activities was taken before the conversion decision. An endoscopy was performed before surgery to evaluate any presence of marginal ulcers and diagnose any complication needing a revision of the anastomoses. The gastric emptying scintigraphy was performed on the last three patients. We also performed it as a control on three patients who underwent surgery during that period and did not present with PEM. The laboratory parameters of albumin, Hb, iron, vitamin B12, folic acid, zinc, calcium, 25 (OH) vitamin D, parathormone, creatinine, glomerular filtration rate (GFR), fasting blood glucose, and HbA1c were evaluated when available during the preoperative period and follow-up.

**Table 1:** Demographics and the symptoms of the patients at the time of conversion surgery and at the last follow-up

										Before conversion				At the last follow-up after conversion			
Case	Primary surgery	Gender	Age	Beginning time of PEM (months)	Time past to revision surgery*	Duration of surgery	Hospital stay after surgery^†^	Date of revision surgery	Last follow-up (month)	Edema	Fatigue	Increased appetite	Stool frequency per day	Edema	Fatigue	Increased appetite	Stool frequency per day
# 1	OATB	M	41	7	3	88 min	5 days	March-17	88 months	Generalized	( +)	( +)	18	( −)	( −)	( −)	2
# 2	OATB	M	64	6	4	96 min	4 days	June-17	85 months	Pretibial	( +)	( +)	7	( −)	( −)	( −)	1
# 3	R-Y TB	F	51	8	4	64 min	6 days	March-18	76 months	Generalized	( +)	( +)	12	( −)	( −)	( −)	0.5
# 4	R-Y TB	F	53	12	5	59 min	3 days	June-18	73 months	Generalized	( +)	( +)	11	( −)	( −)	( −)	1
# 5	R-Y TB	M	56	32	8	48 min	4 days	July-21	37 months	Generalized	( +)	( +)	6	( −)	( −)	( −)	3
# 6	OATB	F	36	34	15	78 min	4 days	November-21	31 months	Pretibial	( +)	( +)	9	( −)	( −)	( −)	0.25
# 7	R-Y TB	F	42	14	19	69 min	3 days	January-22	29 months	Generalized	( +)	( +)	8	( −)	( −)	( −)	2
# 8	R-Y TB	M	33	11	12	44 min	3 days	January-22	29 months	Generalized	( +)	( +)	14	( −)	( −)	( −)	4
#9	OATB	M	65	2	2	62 min	5 days	November-23	7 months	Generalized	( +)	( +)	19	Pretibial	( −)	( −)	5
10	OATB	M	54	Diarrhea without PEM from the beginning	11	75	3	June-24	1 month	No	( +)	( +)	12	( −)	( −)	( −)	2

*OATB* transit bipartition with one anastomosis, *RYTB* transit bipartition with Roux-n-Y configuration, *PEM* protein and energy malnutrition mimicking malabsorption

*The time passed from the first onset of symptoms to conversion

^†^Only post-operative hospital duration is given

### Operational Setup and Preparation for Revision Surgery

All revisions were performed by the same surgeon with experience in various metabolic surgery procedures. The patient lay supine, the surgeon stood on the right, and the monitor was on the patient’s left side for this procedure. Three trocars (5-mm working trocar, 11-mm camera, and 12 mm for stapling) were used for the surgery. Whole intestine lengths were measured as fully stretched from the anti-mesenteric sides. Bowel measurement started from the caecum or Treitz, depending on the availability of the exposure. All the lengths of the limbs were noted. The jejunum was marked with stitches at 100 cm from Treitz. All divisions and anastomoses were performed with vascular (2.5 mm) cartridges.

### The Technique of Conversion of OATB to GJIB

**Fig. 1:**
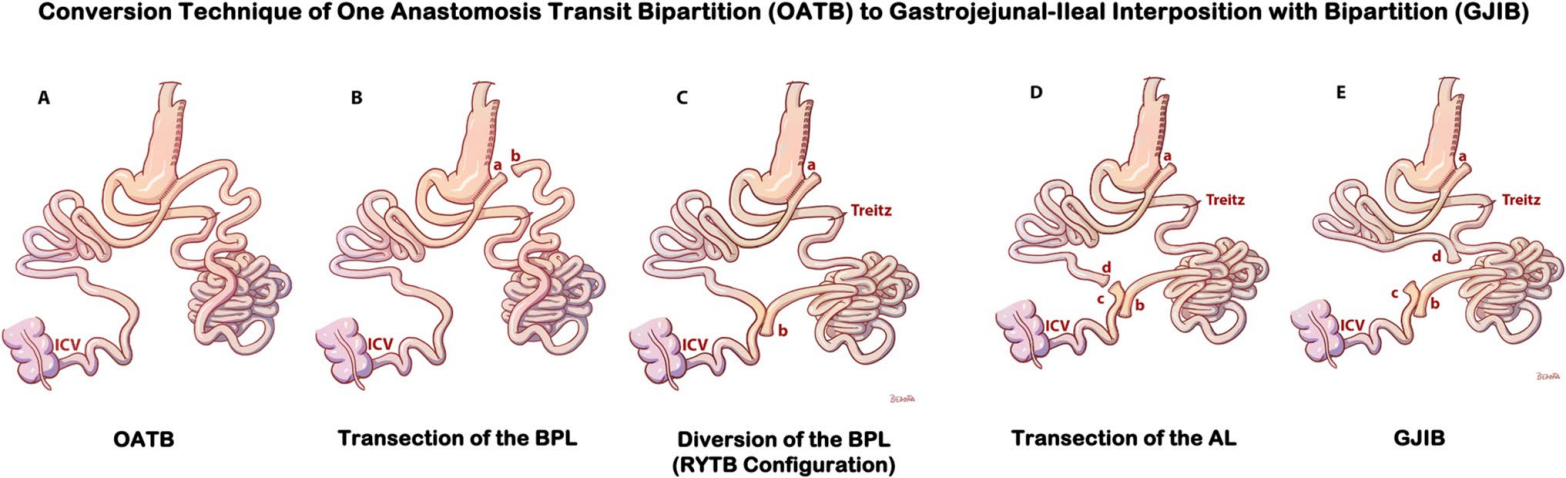
Conversion technique of One Anastomosis Transit Bipartition (OATB) to Gastrojejunal-Ileal Interposition with Bipartition (GJIB). **A** OATB configuration; **B** transection of the Biliopancreatic Limb (BPL) proximal to gastroileostomy anastomosis; **C** side-to-side anastomosis of the BPL to the distal ileum to complete Roux-n-y configuration, the RYTB; **D** transection of the alimentary limb (AL) proximal to the newly formed jejunoileostomy anastomosis; **E** side-to-side anastomosis of the alimentary limb to the proximal jejunum to complete the diversion, the GJIB. ICV, ileocecal valve

Conversion of OATB (shown in Fig. 1A) to GJIB (shown in Fig. 1E) is completed in two steps by performing two anastomoses. The afferent limb is transected proximal to GIA using a vascular (2.5 mm) cartridge (shown as (a) and (b) in Fig. 1B). The transected tip of the afferent limb (shown as “b” in Fig. 1C) is brought next to 30 cm, to 50 cm, to the ileocecal valve (ICV), and re-anastomosed with a vascular (2.5 mm) cartridge. The staple defect is closed with intracorporeal running sutures with polydioxanone 3/0. The alimentary limb is transected proximal to ileoileal anastomosis (shown as (c) and (d) in Fig. 1D). A side-to-side isoperistaltic stapled anastomosis is fashioned with the distal end of the alimentary limb (shown as (d) in Fig. 1E) and the previously marked proximal jejunum at 100 cm from Treitz. The staple opening is repaired and closed with intracorporeal running sutures with polydioxanone 3/0. The mesenteric defect is closed with interrupted stitches using polypropylene 3/0 suture (Prolene®, Ethicon, Somerville, NJ, USA). Figure 1E shows the end configuration of GJIB.

### The Technique of Conversion of RYTB to Gastroileal Interposition with Bipartition (GJIB)

**Fig. 2:**
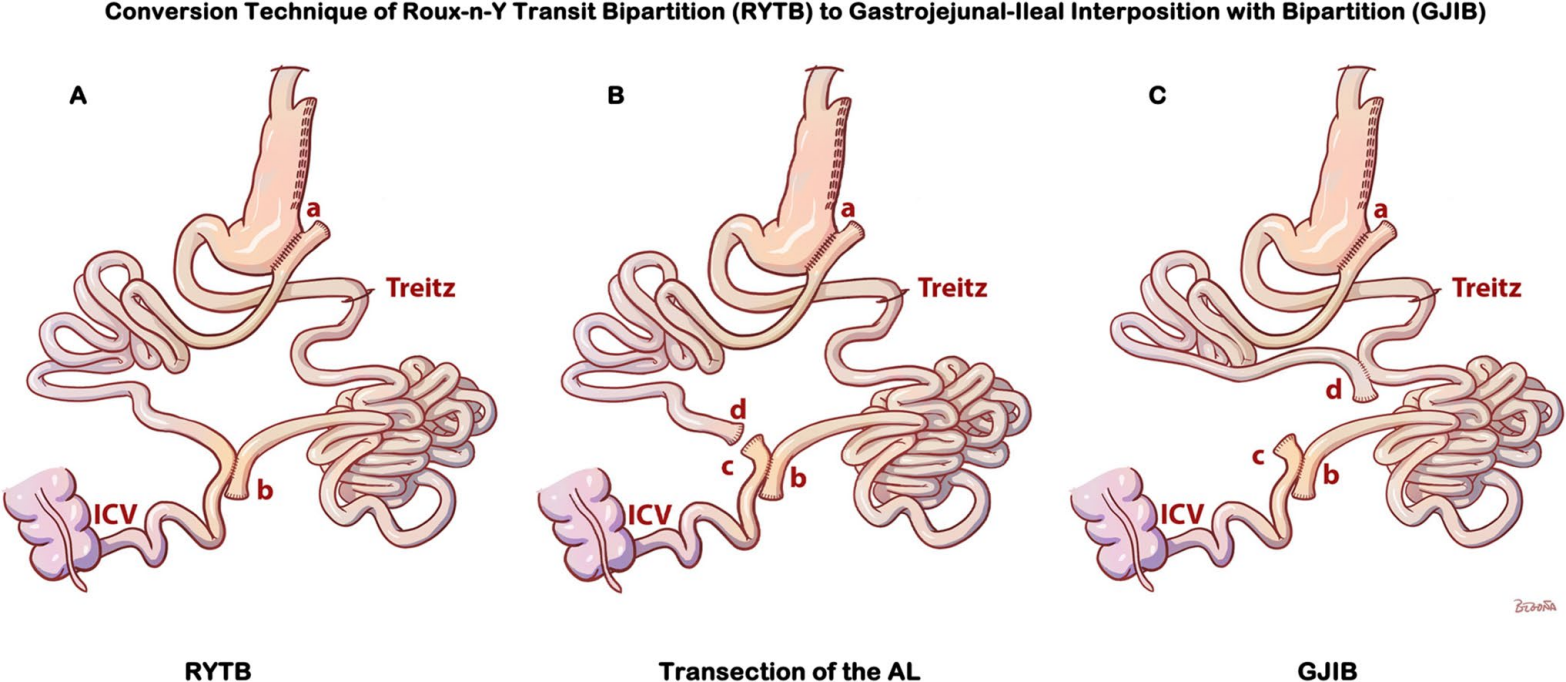
Conversion technique of RYTB to gastrojejunal-ileal interposition with bipartition (GJIB). **A** RYTB configuration; **B** Division of alimentary limb proximal to newly formed jejunoileostomy anastomosis; **C** side-to-side anastomosis of the alimentary limb to the proximal jejunum to complete the diversion, the GJIB. ICV Ileocecal valve

The conversion of RYTB (shown in Fig. 2A) to GJIB (shown in Fig. 2C) is completed in one step by performing one anastomosis. The alimentary limb is transected proximal to ileoileal anastomosis (shown as (c) and (d) in Fig. 2B). A side-to-side isoperistaltic stapled anastomosis is fashioned with the distal end of the alimentary limb (shown as (d) in Fig. 2C) and the previously marked proximal jejunum at 100 cm from Treitz. The staple opening is repaired and closed with intracorporeal running sutures with polydioxanone 3/0. The mesenteric defects are closed with interrupted stitches using polypropylene 3/0 suture (Prolene®, Ethicon, Somerville, NJ, USA). Figure 2C shows the end configuration of GJIB.

### Statistical Analysis

Continuous data were expressed as mean ± SD, median and normal range, and categorical data as numbers and proportions. The Shapiro–Wilks and Kolmogorov–Smirnov tests were used to check the data’s conformity to a normal distribution. The non-normally distributed parameters were shown as median and interquartile (Q3–Q1) ranges where needed. The dependent sample *t*-test was used for normally distributed data, and the Wilcoxon test was used for non-normally distributed data. Data were analyzed using SPSS version 23 (IBM Corp., Chicago, IL).

## Results

**Fig. 3:**
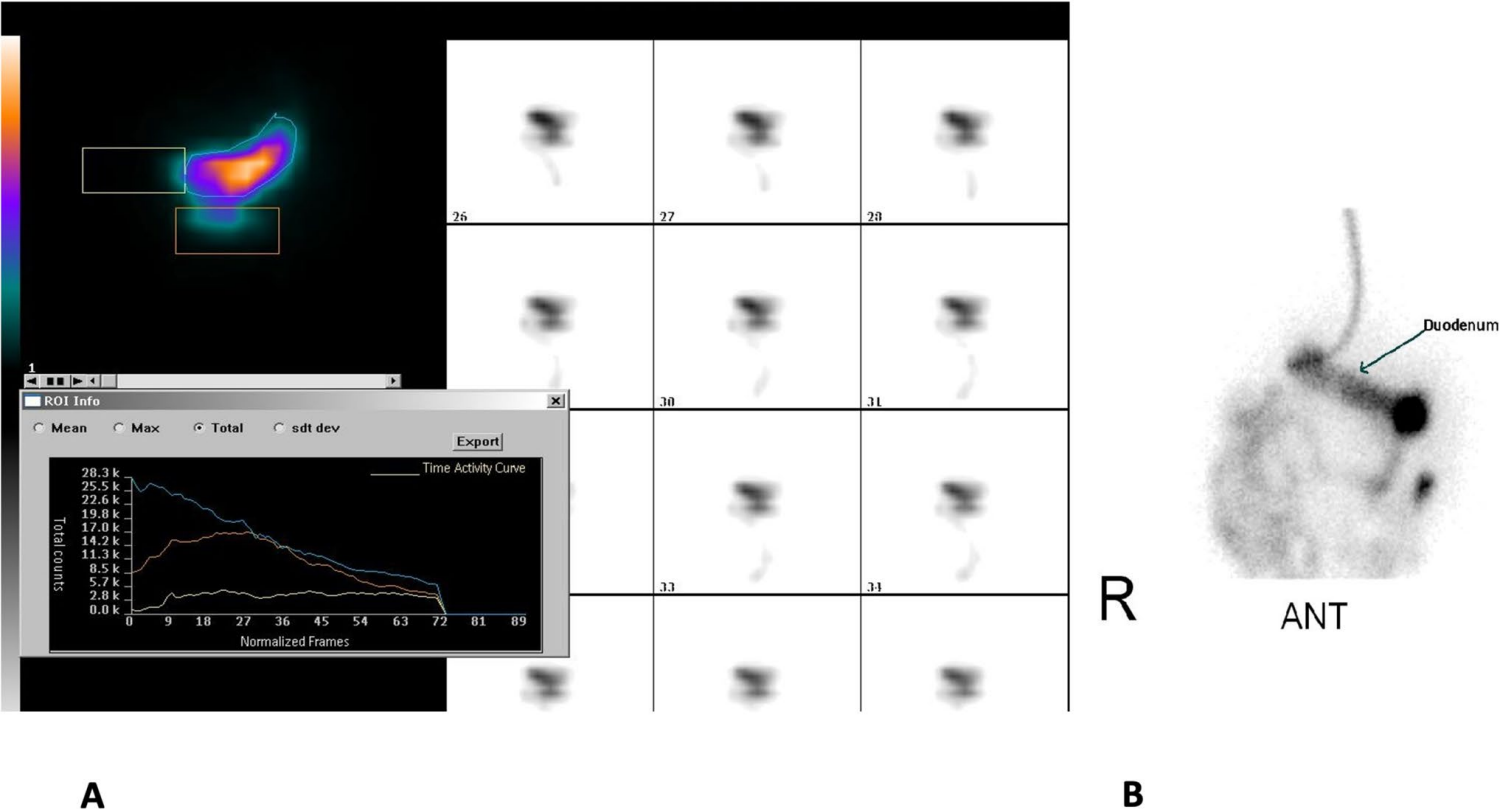
The gastric emptying scintigraphy of a patient. **A** the food passage from the gastroileostomy; the blue line represents the stomach; the orange line represents the gastroileostomy path; and the yellow line represents the pylorus path of radioactively labeled food drainage on a time frame in the graph. **B** Selective cannulation of post-pyloric duodenum for mapping

Five RYTB and five OATB patients who had been converted to GJIB in our center for intractable diarrhea or excess weight loss associated with protein-energy malnutrition (PEM) were included in this retrospective study. We have performed 83 TB/OATB surgeries so far, and six of them had PEM-related symptoms and were included in this study group. Four were female. The mean age was 49.5 ± 9.19 years (33–65). Patients had fatigue despite increased appetite and excess weight loss (the mean body mass index (BMI) of 22.19 ± 1.13 kg/m^2^ (20.72–24.56), and the mean percentage excess BMI loss (%EBMIL) of 123.26 ± 14.85% (102.53–163.55) after initial surgery), generalized or pretibial edema accompanying hypoalbuminemia (mean albumin of 2.7 ± 0.52 mg/dL (1.9–3.8)) with other nutritional parameters, like Hb, iron, vitamin B12, folic acid, zinc, calcium, 25 (OH) vitamin D, parathormone, creatinine, and glomerular filtration rate (GFR), out of normal ranges in some of the patients. Detailed information is provided in Table 1 for demographics and symptomatology, Table 2 for weight changes, and Table 3 for the nutritional parameters. The stool frequency was 11.4 ± 0.71 per day [6–19] before conversion surgery. No anastomotic complication, such as a marginal ulcer or stricture, was observed in preoperative endoscopy, and the anastomosis was approximately 3.5–4 cm. Gastric emptying scintigraphy showed an average of 80% of food passing through the gastro-ileostomy (Fig. 3). The mean time of conversion to GIB was 8 ± 0.71 months [2–19] from the initial surgery, the mean duration of the operation was 67.56 ± 18.38 min, and the mean hospital stay was 4 ± 1.41 days [3–6]. The mean follow-up period was 50.56 ± 57.28 months (1–86 months).

**Table 2:** The average weight parameters of the patients

Prior to primary surgery	Mean ± SD	Range	Time of conversion	Mean ± SD	Range	Last follow-up (avg., 50.5 months)	Mean ± SD	Range
Height (cm)	169.1 ± 7.07	(155–182)	**Lowest weight** (kg)	63.80 ± 2.12^**(1)**^	(52–73)	**Weight at the latest FU** (kg)	80.1 ± 12.73 ^**(1). (2)**^	(68–91)
Weight (kg)	111.3 ± 2.83	(95–132.5)	**TWL** (kg)	47.5 ± 0.71	(33–68.5)	**WG** (kg)	16.30 ± 10.61	(6–35)
BMI (kg/m^2^)	38.9 ± 1.99	(31.74–44.79)	**%TWL** (%)	42.35 ± 0.33	(34.74–51.70)	**%TWL** (%)	27.31 ± 10.1^**(2)**^	(9.90–41.89)
Excess BMI (kg/m^2^)	13.9 ± 1.99	(6.74–19.79)	**%EWL** (%)	123.26 ± 14.85	(102.53–163.55)	**%EWL** (%)	79.88 ± 21.53^**(2)**^	(28.92–133.81)
Ideal weight (kg)	71.66 ± 6.08	(60.06–82.81)	**BMI (**kg/m^2^)	22.29 ± 1.13^**(1)**^	(20.72–24.56)	**BMI (**kg/m^2^)	28.16 ± 2.2^**(1)**^^**,(**^^**2)**^	(22.7–34.3)
Excess weight (kg)	39.64 ± 3.25	(20.18–58.54)	**BMIL (**kg/m^2^)	16.62 ± 0.86	(11.03–23.15)	**BMIL** (kg/m^2^)	10.74 ± 4.19^**(2)**^	(3.76–18.76)
			**%EBMIL** (%)	123.26 ± 14.85	(102.53–163.55)	**%EBMIL** (%)	79.88 ± 21.53^**(2)**^	(28.92–133.81)
						Last FU from conversion surgery (months)*	50.56 ± 57.28	(1–86)

*BMI* body mass index, *TWL* total weight loss compared to initial weight before primary surgery, *%TWL* percentage TWL, *%EWL* percentage excess weight loss, *BMIL* BMI loss compared to initial BMI before primary surgery, *%EBMIL* percentage excess BMIL, *WG* the amount of regained weight after conversion to gastroileal interposition compared to the weight of the patient at the time of conversion, *FU* follow-up

*Time past from the revision surgery to last FU

^(1^^)^*p* ≦ 0.001 compared to the time of initial surgery

^(2^^)^*p* ≦ 0.001 compared to the time of conversion surgery

**Table 3:** The comparison of the nutritional status of the patients before and after conversion from Roux-n-Y transit bipartition (RYTB) and one anastomosis TB (OATB) to gastrojejunal ileal interposition with bipartition (GJIB)

		Time of diagnosis		Time of conversion		*p*-values	Last follow-up		*p*-values
		mean ± SD	range	mean ± SD	range		mean ± SD	range	
Albumin	(3.5–5.2 mg/dl)	2.1*	(2.4–1.9)*	2.7 ± 0.52	(1.9–3.8)	*p* = 0^**(1)**^	3.9 ± 0.42	(3.1–4.5)	*p* = 0^**(1)**^^**,(**^^**2)**^
Hb	(14–18 g/dl)	10.8 ± 1.58	(8.9–13.4)	10.6 ± 0.88	(9.2–11.7)		13.3 ± 1.57	(10.7–16.4)	*p* = 0^**(1)**^^**,(**^^**2)**^
Iron	(65–176 µg/dl)	57.8 ± 25.15	(24–100)	52.6 ± 14.85	(36–78)		64.1 ± 16.34	(44–94)	N/A
VitaminB12	(211–911 ng/L)	272.2 ± 108.24	(163–487)	332.2 ± 90.4	(211–482)		423.5 ± 122.21	(236–624)	*p* = 0.006^**(1)**^
Folic acid	(2.6–12.2 pg/L)	7.2 ± 5.07	(3.1–20)	7.8 ± 3.02	(3.3–14.1)		10.7 ± 2.20	(7.6–14.1)	N/A
Zinc	(50–150 µg/dl)	68.3 ± 16.38	(54–97)	56 ± 10.8	(44–74)		103.9 ± 18.85	(78–134)	*p* = 0.04^**(1)**^
Calcium	(8.6–10.5 mg/dl)	8.6 ± 0.41	(8.0–9.2)	8.7 ± 0.39	(8.1–9.6)		9.1 ± 0.50	(8.3–10.2)	*p* = 0.009^**(1)**^; *p* = 0.025^**(2)**^
25(OH)-vitamin D	(> 30 ng/ml)	13 ± 6.5	(5.4–24)	18.1 ± 4.22	(12.5–24)		29.9 ± 7.68	(19–43)	*p* = 0.001^**(1)**^; *p* = 0^**(2)**^
PTH	(15–65 ng/L)	57 ± 22.96	(17.2–88)	63 ± 12.28	(42–77)		56.3 ± 12.98	(33–77)	N/A
Creatinine	(0.67–1.27 mg/dl)	0.81 ± 0.28	(0.51–1.44)	0.9 ± 0.28	(0.52–1.31)		1 ± 0.08	(0.85–1.14)	N/A
GFR	(> 90 ml/min/1.73 m^2^)	88.1 ± 16.05	(51.8–113)	82.6 ± 13.63	(61.3–110)		89.4 ± 12.29	(65–107)	N/A
FBG	(< 100 mg/dl)	94.3 ± 16.95	(72–129)	93.7 ± 14.75	(77–115)		95.5 ± 10.74	(76–113)	N/A
HbA1c		5.75*	(6–5.3)*	5.8*	(6–5.4)*		5.4*	(6–5.3)*	N/A

*PTH* parathormon, *GFR* glomerular filtration rate, *FBG* fasting blood glucose

*The non-normally distributed parameters are shown as median and interquartile ranges (Q3–Q1)

^(1^^)^Compared to the time of initial surgery

^(2^^)^Compared to the time of conversion surgery

The bowel lengths were measured in all patients in this study. The average total bowel length was 854.5 ± 205.06 cm (735–1080), and the excluded bowel length percentage (Exl.BL%) was reduced from a median of 72.4 ± 3.18% to an average 13.4 ± 7.3% after converting to GIB configuration (*p* = 0.005). The details of the bowel measurements are given in Table 4. All patients were discharged without any surgical complications or mortality.

**Table 4:** The limb lengths of patients’ before and after conversion

Limb lengths of primary surgery (OATB or RYTB)					Revisional GJIB configuration			
	Alimentary limb (Roux limb + CL) (cm)	BPL (cm)	TBL (cm)	Exl.BL%	Neo-alimentary limb (Roux limb + CL) (cm)	Neo-BPL (cm)	TBL (cm)	Neo-Exl.BL%
Mean ± SD	280 (± 53.03)	620 (± 173.24)	854.5 (± 205.06)	72.4%*	732,5 (± 106.7)^**(1)**^	100*^,^^**(**^^**1)**^	842 (± 212.13)	12.35%*^,^^**(**^^**1)**^
Min	185	480	725	66.2%	620	100	720	10%
Max	325	785	1080	78.4%	900	250	1080	23.2%

To simplify, the Roux limb and CL of the RYTB configuration were totally collected under the name of the alimentary limb which corresponds to the CL in OATB

*GJIB* Gastrojejunal Ileal Interposition with bipartition, *RYTB* Roux-n-y transit bipartition, *OATB* one anastomosis transit bipartition, *AL* the sum of the Roux limb and the common limb in RYTB configuration and the CL in OATB configuration, *CL* common limb, *BPL* biliopancreatic limb, *TBL* total bowel length, *Exl.BL%* excluded bowel length percentage, *Neo-Roux L* the new Roux limb length in revisional gastroileal interposition with bipartition configuration, *Neo-CL* the length of the common limb in the new configuration, *Neo-BPL* the length of the biliopancreatic limb in the new configuration, *Neo-Exl.BL%* the new excluded bowel length percentage

^*^Related values represent the median of non-normally distributed parameters

^(1^^)^*p* ≦ 0.005 compared to the initial surgery

The postoperative mean BMI increased to 28.16 ± 2.2 kg/m^2^ (22.7–34.3) (*p* = 0.001), and the mean %EBMIL decreased to 79.88 ± 21.53% (28.92–133.81) (*p* = 0.001) on the last follow-up after conversion surgery. Mean albumin levels increased to 3.9 ± 0.42 (3.1–4.5) mg/dl (*p* = 0), and the stool frequency decreased to 2.1 ± 2.12 (0.3–5) daily (*p* = 0). Only one patient still had pretibial edema and fatigue at the last follow-up, which had a comparatively short follow-up of 5 months. All patients were diabetic before initial surgery and had a control or improvement of diabetes at the conversion time with a median HbA1c of 5.8% (6–5.4). The glycemic control was preserved after the conversion, with median HbA1c of 5.4% (6–5.3) in the last follow-up with no significant difference.

## Discussion

This study introduces a novel surgical technique to address protein-energy malnutrition (PEM) and intractable diarrhea following Transit Bipartition (TB). Patients experiencing persistent malnutrition, hypoalbuminemia, and excessive weight loss despite conservative management underwent conversion to Gastro-Jejunal Ileal Interposition (GJIB). This approach reintegrates functionally excluded small bowel segments while preserving the metabolic benefits of bipartition.

PEM after TB is rare but has been reported in a few cases, often associated with excess weight loss and diarrhea [3–5]. The etiology of these complications has been reportedly linked to excessive nutrient bypass due to a dominant new gastric outlet (wide gastroileostomy) [3, 4] or a short alimentary limb following the anastomosis [5], leading to accelerated transit and reduced absorption. While studies indicate that GIA diameters vary widely from 2.5 to 6 cm in diameter, the precise role of anastomotic size in malabsorption remains controversial [2, 7–11]. The details on the limb lengths, gastroileostomy anastomotic diameters, and staplers used in the other reports are summarized in Table 5 [2–6, 8–19].

**Table 5:** A summary of the operative details of previous reports

Author	Year	Type	Stapler/GIA width	GIA position	Nutritional limb length (cm)	TBL measurement
Santoro [2]	2012	TB	?/3–4 cm	Posterior gastric wall	260	N/A
Salama [19]	2017	OATB	45 mm/?	Posterior gastric wall	300	N/A
Mahdy [16]	2016	OATB	45 mm/3 cm	Anterior gastric wall	250	N/A
Aslan [12]	2018	TB/OATB	45 mm/?	Posterior gastric wall	250/300	N/A
Topart [29]	2020	TB	30 mm/?	?	250	N/A
Bilecik [13]	2019	TB	?/3–4 cm	?	260	N/A
Karaca [15]	2020	TB	35 mm/3.5 cm	Posterior gastric wall	260	N/A
Kermanserawi [3]	2020	OATB	?/3 cm	Anterior gastric wall	250	N/A
Mahdy [17]	2020	OATB	45 mm/3 cm	?	300	N/A
Topart [11]	2020	TB	30 mm/?	?	250	N/A
Al [7]	2021	TB	45 mm/3.5 cm	?	260	N/A
Reiser [6]	2021	TB	45 mm/3.5–4.5 cm	?	250–300	N/A
Gulaydin [9]	2022	TB	60-mm linear/ > 5.5 cm, 25 mm circular/ < 2.5 cm	Lateral edge of the gastric wall	250	N/A
Robert* [10]	2022	TB	< 2-cm hand sewn	?	250	N/A
Tarnowski [4]	2022	OATB	?/?	Anterior gastric wall^†^	300	N/A
Demir [14]	2023	TB/OATB	45 mm/ > 4 cm	Posterior gastric wall	250/300	N/A
Ribeiro [18]	2023	OATB	?/3–4 cm	?	250/300	N/A

Nutritional limb comprises of the alimentary limb and common limb in TB and the post anastomotic limb in OATB. Some of the authors reported an average assumptive diameter of the GIA but did not mentioned the length of stapler used. Others gave the length of the stapler only. The reported GIA diameters ranged between < 2 and 6 cm. None of the papers reported any TBL length measurement; question marks (?) imply that related information was not reported in the article

*GIA* gastroileal anastomosis, *TBL* total bowel length, *TB* transit bipartition, *OATB* one anastomosis transit bipartition

*Report of a case of robotic TB

^†^The anastomosis positioning was not reported in the article, but the figure shows it is placed in the anterior gastric wall

Our gastric emptying scintigraphy demonstrated that food predominantly traversed the anastomotic route in symptomatic and asymptomatic patients with similar anastomoses. This suggests that it is not the size of the gastroileostomy anastomoses that leads to PEM and diarrhea but the excessive exclusion of the unknown length of the proximal bowel in some patients**.**

Total bowel length (TBL) variations significantly impact outcomes following bariatric surgery, influencing weight loss and nutritional status [20]. The length of bypassed intestinal segments is crucial in determining postoperative efficacy and complications, as demonstrated in RYGBP and one anastomosis gastric bypass [21]. Stool alterations, including severe diarrhea, excessive flatulence, and foul-smelling stools, frequently occur after malabsorptive surgeries, requiring treatment or revision in cases where PEM becomes life-threatening [22, 23].

A key factor contributing to variability in surgical outcomes is the broad range of TBL among individuals, which exceeds previously assumed norms. Studies assessing anthropometric parameters and TBL have identified extreme differences, with patient TBLs ranging from 205 to 1049 cm [24, 25]. Tacchino et al. [24] found that 20% of patients had TBLs below 400 cm or above 800 cm, which could impact common limb length and influence weight loss and nutritional stability following RYGBP.

Eagleston and Nimeri [21] simulated different bariatric surgery scenarios using standard limb lengths in patients with varying TBLs of 500 cm, 700 cm, or 900 cm. Their findings demonstrated that some patients developed PEM, while others failed to meet weight loss expectations based on their TBL and procedure type. These variations highlight the necessity of individualized surgical planning to optimize patient outcomes and minimize complications.

An essential aspect in the planning of this revisional surgery is to decide whether to preserve bipartition and beneficial metabolic effects or convert to a sleeve gastrectomy. Preserving metabolic effects is important because disconnecting the ileum from the stomach eliminates early ileal stimulation, reducing hormonal benefits that enhance glucose metabolism and weight control. Converting TB to a sleeve gastrectomy reverses the anatomical changes responsible for TB’s metabolic benefits and is expected to increase the likelihood of weight regain and metabolic decline. In contrast, by restructuring proximal bowel segments, GJIB significantly enhances absorptive capacity, restoring protein and micronutrient levels without compromising metabolic control. Another advantage of GJIB is its maintenance of endoscopic access as in all bipartition techniques, unlike duodenal switch procedures that eliminate upper gastrointestinal endoscopic access.

Almahmeed, Pomp, and Gagner [26] had to revise two patients for developed significant malnutrition, who had undergone duodenal switch (DS) as the primary surgery as they had BMIs greater than 50. Both patients suffered from severe weight loss (WL), diarrhea, and PEM. The nadir BMIs of the patients prior to revision were 25 kg/m^2^ (representing 81 kg of WL) and 21 kg/m^2^ (representing 88 kg of WL), respectively. They both underwent partial common limb elongations as a first step, which did not halt further weight loss and PEM. Ultimately, both patients had ileal interpositions, one to the duodenum and the other to the proximal jejunum (30 cm from the Treitz ligament), significantly enhancing the intestine’s absorptive capacity. However, both patients regained a limited amount of excess weight while maintaining a significant portion of their weight loss, with BMIs rising to 29 kg/m^2^ [26].

In comparison, the average BMI of our patients increased from 22.29 to 28.16 kg/m^2^, preserving the metabolic and weight loss effects of the Transit Bipartition (TB) on one hand while improving PEM and hypoalbuminemia on the other.

Dapri [27] also reported a total reversal of one DS patient for intractable PEM and diarrhea, with a total weight loss of 78.5 kg and a BMI decrease from 54 to 24.5 kg/m^2^. He deconstructed duodenoileostomy and re-anastomosed the duodenal stump with post-pyloric duodenum, reconfiguring a normal antro-pyloro-duodenal passage, and completing with an ileo-ileostomy, re-establishing the original intestinal anatomy. This patient had retained only the sleeve component and lost the hindgut stimulation while correcting malabsorption. This is a perfect example of how the results of a sleeve-alone procedure will be markedly different from a sleeve plus ileal interposition. This patient gained 47.5 kg in 6 months, and her BMI rose to 42 kg/m^2^. This is significantly different from our patients’ weight regain levels. In their case, Almahmeed, Pomp, and Gagner [27] also did not experience such weight regain in which they interposed the ileal segment to the duodenum, enabling the food to traverse the whole length of the small bowel. These authors also emphasized that there wasn’t a greater weight regain despite the loose sleeve pouch [27]. Various other authors also proposed and used ileal interposition to correct severe PEM related to malabsorption [28, 29].

Our results are consistent with previous reports regarding the reversal or revision of duodenal switch surgeries for severe protein-energy malnutrition (PEM) and diarrhea related to malabsorption. Post-revision outcomes in our study support the effectiveness of transposing the ileal segment proximally as in GJIB. After surgery, the excluded bowel length decreased from 72.4 to 13.4%, reinforcing the role of bypassed intestinal length in PEM pathogenesis. Albumin levels rose from a median of 2.7 to 3.9 mg/dL, indicating a significant improvement in protein absorption. In contrast, BMI increased from 22.29 to 28.16 kg/m^2^, signifying effective weight stabilization while maintaining metabolic improvements.

Converting the TB to a sleeve in patients with PEM and excess weight loss corrected the malnutrition and diarrhea; however, none of the authors mentioned these patients’ metabolic and weight loss outcomes in their reports [3–5]. Unlike total anatomical reversal, which may cause complete metabolic regression and weight regain [27], we preferred to perform GJIB, which selectively restores absorption without negating bipartition effects, aligning with the previous experiences**.** This selective restoration of absorption was pivotal for achieving optimal patient outcomes.

In conclusion, though rare, the occurrence of PEM following TB highlights the importance of total bowel length and its effect on nutrient absorption**.** While a simple reversal to sleeve gastrectomy may seem like a straightforward solution, it comes with the risk of metabolic deterioration and possible weight regain. GJIB represents a more sophisticated approach, addressing PEM while maintaining the metabolic benefits of TB. Future studies should further assess individualized surgical strategies based on variations in total bowel length to optimize patient outcomes. However, GJIB may be a highly safe and predictable surgical intervention that prevents malabsorption while functionally excluding the foregut and stimulating the hindgut, improving insulin resistance using multiple mechanisms.

This study has significant limitations. Due to the retrospective nature of the study, we reported only our limited number of cases, gathering a small sample size. As we have significant experience with Duodenal Switch (DS) and Duodenoileal Interposition (DII) besides TB and have revised some of our DS patients to DII for PEM, we are familiar with the effect of hindgut stimulation on the control of excess weight regain and metabolic outcomes. So, we preferred to transpose the ileal segment, which was not technically demanding. However, this led to the failure to do sleeve-only revisions, and we could not have a cohort to compare. Further research is needed to refine patient selection criteria and validate these findings in larger cohorts, possibly in a multicenter study, comparing both techniques on feasibility and outcomes.

## Data Availability

No datasets were generated or analysed during the current study.
